# Gene Knockout Study Reveals That Cytosolic Ascorbate Peroxidase 2(OsAPX2) Plays a Critical Role in Growth and Reproduction in Rice under Drought, Salt and Cold Stresses

**DOI:** 10.1371/journal.pone.0057472

**Published:** 2013-02-28

**Authors:** Zhiguo Zhang, Quian Zhang, Jinxia Wu, Xia Zheng, Sheng Zheng, Xuehui Sun, Quansheng Qiu, Tiegang Lu

**Affiliations:** 1 Biotechnology Research Institute, Chinese Academy of Agricultural Sciences/National Key facility for Gene Resources And Genetic Improvement, Beijing, China; 2 MOE Key Laboratory of Cell Activities and Stress Adaptations, School of Life Sciences, Lanzhou University, Lanzhou, China; RIKEN Plant Science Center, Japan

## Abstract

Plant ascorbate peroxidases (APXs), enzymes catalyzing the dismutation of H_2_O_2_ into H_2_O and O_2_, play an important role in reactive oxygen species homeostasis in plants. The rice genome has eight *OsAPXs*, but their physiological functions remain to be determined. In this report, we studied the function of *OsAPX2* gene using a T-DNA knockout mutant under the treatment of drought, salt and cold stresses. The *Osapx2* knockout mutant was isolated by a genetic screening of a rice T-DNA insertion library under 20% PEG-2000 treatment. Loss of function in *OsAPX2* affected the growth and development of rice seedlings, resulting in semi-dwarf seedlings, yellow-green leaves, leaf lesion mimic and seed sterility. *OsAPX2* expression was developmental- and spatial-regulated, and was induced by drought, salt, and cold stresses. *Osapx2* mutants had lower APX activity and were sensitive to abiotic stresses; overexpression of *OsAPX2* increased APX activity and enhanced stress tolerance. H_2_O_2_ and MDA levels were high in *Osapx2* mutants but low in *OsAPX2*-OX transgenic lines relative to wild-type plants after stress treatments. Taken together, the cytosolic ascorbate peroxidase OsAPX2 plays an important role in rice growth and development by protecting the seedlings from abiotic stresses through scavenging reactive oxygen species.

## Introduction

Reactive oxygen species (ROS) control various signaling pathways in plants involved in stress and pathogen responses, photosynthesis, programmed cell death, hormonal action, growth and development [1], [2]. ROS might cause cellular injury through reacting with biological compounds [3]. ROS damage is one of the major mechanisms underlying the biotic and abiotic stresses including drought, high light, wounding, salt, or pathogen infection [4], [5], [6].

Cells have evolved highly regulated mechanisms to maintain a balance between ROS production and destruction [7]. Ascorbate peroxidases (APX) are antioxidant enzymes functioning in converting H_2_O_2_ into H_2_O and O_2_ [8]. Studies have shown that APXs play an important role in removing ROS in plants [1]. In *Arabidopsis*, cytosolic APXs (cAPXs) are critical for cellular H_2_O_2_ homeostasis and play an important role in oxidative protection of chloroplasts under abiotic stresses, including high light, heat, methyl viologen and drought stress [9], [10]. *Arabidopsis* APX1 is important for plant growth and development since mutation of *APX1* leads to the accumulation of H_2_O_2_, inhibition of plant growth and photosynthesis, delay of flowering, and enhanced protein oxidation under high light [10]. *APX2* expression is induced under high light, heat stresses and wounding conditions [11], [12]. APX2 is also critical for drought tolerance in *Arabidopsis* [13]. Thylakoid-bound APXs (tAPXs) are essential for photosynthetic activity and photoprotection under photooxidative stress in *Arabidopsis* [14].

Rice genome contains eight *APX* genes. OsAPX1 and OsAPX2 are in the cytosol, OsAPX3 and OsAPX4 in peroxisomes, OsAPX6 in mitochondria, and OsAPX5, OsAPX7 and OsAPX8 in chloroplasts [15]. The expressions of *OsAPX1* and *OsAPX2* are developmentally regulated [16]. The silencing of *OsAPX1* and *OsAPX2* genes individually resulted in strong effect on plant development, producing semi-dwarf phenotype [17]. The expressions of *OsAPXs* are also modulated by environmental stimuli, such as drought, high light, high temperature, salt stress, pathogen attacks and exogenous ABA [15], . Overexpression of *OsAPX1* enhances tolerance to chilling at the booting stage in rice [18]; however, overexpression of *OsAPX2* improves salt tolerance in transgenic *Arabidopsis* and *Medicago sativa* [19], [20].

Currently, the studies on rice APXs are using either overexpression or RNAi techniques, no knockout mutants have been used to explore the function of APXs in rice. The major concern regarding RNAi is that it generates knockdown mutants which can have different phenotypes compared with the knockout mutants. Another drawback of RNAi is its “off target” silencing, in which the genes with similar sequences are non-specifically silenced [21]. Therefore, it is necessary to apply insertion mutagenesis, which generates site specific null mutants, to confirm the observations obtained from the RNAi analysis [22]. On the other hand, there is only single abiotic stress (ie. either drought, or salt or cold stress) applied to elucidating the APX function in the previous studies [18], [19], [20]. In this study, we isolated a loss-of-function rice mutant *Osapx2* through genetic screening of a T-DNA insertion population. The function of OsAPX2 in drought, salt and cold stresses tolerance is extensively investigated at seedling and reproductive stages.

## Materials and Methods

### Mutant Screening

We have screened more than 100,000 T-DNA insertion rice lines generated in our laboratory with 20% PEG2000 (simulation of drought stress) [23]. Thirty seeds from each line were sown in petri dish (10 cm in diameter) with 20 ml sterile distilled water. After two days of germination, seedlings were treated with 20% PEG2000 for 48 h and recovered in water for another 48 h. One T-DNA insertion line was found to produce small seeds and exhibited severe inhibition in root elongation and seedling growth compared with the wild-type control (Nipponbare).

### Anatomical Observations

Floral structures of the mutant, complementation and wild-type plants were observed under an optical microscope before flowering. Pollen viability was determined according to staining gradation under a microscope with 1% I2-KI solution and nipping into pieces with forceps to spill out pollen grains [24].

### Identification of the *Osapx2* Mutant

Genomic DNA flanking the T-DNA left border was amplified using polymerase chain reaction (PCR) walking with nesting-specific primer pairs according to the method described by Peng et al. [25]. The specific primers for the T-DNA left border were LB1 (5′-CGATGGCTGTGTAGAAGTACTCGC-3′) and LB2 (5′-GTTCCTATAGGGTTTCGCTCATGTGTTG-3′). The specific primers for the adaptor were APR1 (5′-GGATCCTAATACGAGTCACTATAGCGC-3′) and APR2 (5′-CTATAGCGCTCGAGCGGC-3′). PCR products were directly sequenced. The T-DNA insertion site in the mutant was defined by NCBI BLAST of the rice genome database (http://www.ncbi.nlm.nih.gov/Blast/) using the rescued flanking sequences, and BLAST results showed that the T-DNA was inserted in the fourth intron of *OsAPX2*.

### Genotyping of *Osapx2* with PCR

Genotyping of *Osapx2* in T_1_ segregating population was performed by PCR using the following primers: P1 (5′-AACTTCCCATCCTCTCCTAC-3′), P2 (5′- CAAAGAAGGCGTCCTCATC-3′) and LB2 (5′-GTTCCTATAGGGTTTCGCTCATGTGTTG-3′) (Figure 3-A). More than 100 individual plants were analyzed. One µg of DNA was used as template with primers mixture (1 µM of P1, P2 and LB2, respectively) in a 20 ml reaction system. PCR was conducted with an initial step of 94°C incubation for 3 min and 30 cycles of 94°C for 30 s, 54°C for 30 s, and 72°C for 1 min.

### Complementation and Over Expression of *OsAPX2*

*OsAPX2* full-length cDNA (905 bps) was amplified by PCR with the primers OP1 containing a *Sal*I digestion site (5′- cgGTCGACGTGAGTTGAGTTGGGGATTG-3′) and OP2 containing a *Pst*I digestion site (5′-gctCTGCAGCCACGACAGTCTTGATCGTAC-3′). The PCR product was cloned into the pEASY-Blunt simple cloning vector (Transgen Biotech) and sequenced. For complementation construct, full-length cDNA of *OsAPX2* was cloned into the vector carrying 2.1-kb *OsAPX2*-promoter (from -2111 bp to -1 bp) to generate p*OsAPX2*::*OsAPX2*. For the over expression construct, cDNA were excised from the vector by *Sal*I and *Pst*I digestion and subcloned into pCAMBIA-23A. In pCAMBIA-23A, *OsAPX2* was downstream of the *Actin* promoter. The constructs were introduced into *Agrobacterium tumefaciens* (stain EHA105) by electroporation. *Agrobacterium*-mediated transformation was performed using vigorously growing calli derived from mature embryos of *Osapx2* mutant and wild-type Nipponbare following an improved protocol described by Yang et al. [26].

### RT-PCR and Quantitative RT-PCR

For molecular analysis of *Osapx2* mutants, total RNA was isolated using TRIzol solution (Invitrogen) from leaves of wild-type and *Osapx2* plants. For expression analysis of *OsAPX2*, total RNA was extracted from different tissues, including roots, leaves, blade sheath, blade ears, flowers, apical meristems and internodes. Total RNA from each sample was reversely transcribed with oligo (dT) primer and PrimeScript RT Enzyme (TaKaRa) according to the manufacturer’s instructions. For real time PCR (RT-PCR), the PCR primers for amplifying *OsAPX2* were RT-1 (5′-AACTTCCCATCCTCTCCTAC-3′) and RT-2 (5′-CTCTCCTTGTGGCATCTTCC-3′). PCR conditions were as follows: preincubation at 94°C for 2.5 min, then 30 cycles at 94°C for 30 s, 54°C for 30 s, and 72°C for 1 min. The rice *Actin* gene, amplified with primers Actin1 (5′-TGCTATGTACGTCGCCATCCAG-3′) and Actin2 (5′-AATGAGTAACCACGCTCCGTC-3′), was selected as an internal standard to normalize the expression of *OsAPX2*. Quantitative real-time PCR (RT-qPCR) was performed on the iQ5 Multicolor Real-Time PCR Detection System (Bio-Rad) with real-time PCR Master Mixture (SYBR Green Mix). The primers for *OsAPX2* were *OsAPX2*F (5′-CATCCTCTCCTACGCCGAC-3′) and *OsAPX2*R (5′-CCTTCAGGAGGAGGCTCAG-3′). The primers for *OsAPX1* were *OsAPX1*F (5′- GCCGATTTCTACCAGCTTCTTG-3′) and *OsAPX1*R (5′- AGGGTGTGACCGCCAGAGA-3′). The rice *Actin* gene was amplified with primers ActinF (5′-TGCTATGTACGTCGCCATCCAG-3′) and ActinR (5′-AATGAGTAACCACGCTCCGTCA-3′). RT-qPCR was performed in triplicate for each individual line and threshold cycle values were quantified by RT-qPCR by calculating means of normalized expression using the relative quantification method [27].

### Expression Profile of *OsAPX2* under Stress Conditions

Rice seeds were surface sterilized for 5 min with ethanol (75% v/v) and 10 min with commercially diluted (1∶3 v/v) NaOCl twice, followed by 3 to 5 rinses with sterile distilled water. Germination was carried out for 72 h on sterile Murashige and Skoog (MS) medium in the dark at 28°C day/25°C night temperatures, 12 h light/12 h dark cycle, and 50% humidity. Two-week-old seedlings were used for abiotic stress treatments. For the drought treatment, seedlings were transferred to Whatman 3 MM paper in a sterile petri dish for 0, 0.5, 1, 2 and 4 h. For the cold treatment, 4°C for 0, 0.5, 1, 2 and 4 h; control plants were harvested at the same time. For the salt treatment, 0 mM, 50 mM, 100 mM, 150 mM and 250 mM NaCl for 1 h. *OsAPX1* expression in *Osapx2* mutant was analyzed under normal and stress conditions according to the method described as above, except that seedlings of wild-type and *Osapx2* were harvested after 1 h treatment for drought, cold and salt (150 mM NaCl) treatments.

### Histochemical GUS Assay

Plant material was prefixed in 90% acetone for 1 hour at –20°C, washed twice with staining buffer without X-Gluc and infiltrated with staining solution (100 mM sodium phosphate buffer pH 7.0, 10 mM sodium EDTA, 5 mM potassium-ferrocyanide, 5 mM potassium-ferricyanide, 0.1% Triton X-100, 0.1 mg/ml chloramphenicol, 2 mM X-Gluc) under vacuum for 20 min and incubated at 37°C for 24 h. Chlorophyll was extracted by passing through increasing concentrations of ethanol. The samples were cleared as described by Malamy & Benfey [28].

### Evaluation of Abiotic Stresses Tolerance at the Seedling Stage

Seeds of *Osapx2* mutant and its segregated wild-type plants were germinated and grown in pots filled with vermiculite in a greenhouse at 30°C (light) and 22°C (dark) under 16-h-light/8-h-dark conditions. Then seedlings with four leaves were treated with different stresses. Salinity treatment was performed by immersed the pots into 200 mM NaCl for 10 days. For low temperature treatment, plants were transferred to a 16°C incubator for 14 days. For drought treatment, plants were withheld water for 6 days. For stresses tolerance evaluation of *OsAPX2*-overexpressing plants, seedlings of *OsAPX2*-OX and wild-type with four leaves were treated and time courses were extended to 18 days for salinity treatment, 18 days for low temperature treatment and 10 days for drought treatment. Phenotype changes of treated plants were carefully investigated and photographed. Contents of relative water, chlorophyll, H_2_O_2_ and malondialdehyde (MDA) were measured immediately after stress treatments. Each treatment was repeated three times.

### APX Activity Assays

Five-leaf seedlings grown in the half-strengthened MS liquid medium were used. For salt treatment, wild-type, *Osapx2* and *OsAPX2*-OX plants were treated with 150 mM NaCl for 24 h; for cold treatment, plants were transferred to a 10°C incubator for 24 h; for drought treatment, plants were transferred to dry petri dishes for 2 h.

APX activity was determined according to the method of Chen and Asada (1989) with minor modifications [29]. The 200 mg tissues were homogenized in 50 mM sodium phosphate buffer (pH 7.0) containing 1 mM EDTA and 5 mM ascorbate. The 1-ml reaction mixture was composed of 50 mM sodium phosphate buffer (pH 7.0) containing 0.1 mM EDTA and 0.5 mM ascorbate, 1.54 mM H_2_O_2_, and 0.1 ml of enzyme extracts. The oxidation of ascorbate was determined by the decrease in the absorbance at 290 nm. APX activity was expressed as micromole of ascorbate oxidized per minute.

Protein content was determined using bovine serum albumin as a standard, according to the method of Bradford (1976) [30]. All enzyme activities were calculated per milligram of protein per minute.

### Relative Water Content Assay

Relative water content of leaves was measured on treated and control plants. The fresh weight (FW) was measured immediately after these materials were taken. All leaves were macerated in deionized water overnight at 4°C and the leaves turgid weight (TW) was recorded. Finally these leaves dehydrated in drying cabinet at 70°C until the constant weight for 8 h and obtained the dry weight (DW). Relative water content was calculated using the equation: RWC (%) = (FW−DW)/(TW−DW) ×100% [31].

### Chlorophyll Content Assay

Total chlorophyll content was determined spectrophotometrically according to the method described by Arnon (1949) [32]. The 300 mg leaves were grinded into powder with liquid nitrogen and then were transferred to a 15 ml Falcon tube. Add 5 ml of 80% acetone to the tube mix thoroughly and stand in dark overnight. Centrifugation was performed at 4°C for 15 min (3,000 rpm). Supernatant was transferred to a new centrifuge tube and measure the absorbance of chlorophyll (hereafter termed A) using spectrophotometry. The chlorophyll concentrations are calculated as follows (use 80% acetone as a blank control). Ca+b (mg/g) = [8.02×A_663_+20.20×A_645_]×V/1000×W. Where V = volume of the extract (ml); W = Weight of fresh leaves (g).

### Hydrogen Peroxide Determination

Determination of H_2_O_2_ levels were carried out according to the method of Dagmar (2001) [33]. Briefly, 500 mg of leaves were homogenized in a cold mortar with a pestle and 0.2 g silicon dioxide in pre-cooled acetone (5 ml) and the homogenate was centrifuged at 12,000 g for 5 min. One milliliter of the supernatant was mixed with 0.1 ml of 5% Ti(SO_4_)_2_ and 0.2 ml of 19% ammonia. After precipitate was formed the reaction mixture was centrifuged at 12,000 g for 5 min. The resulting pellet was dissolved in 3 ml of 2 M H_2_SO_4_ and the absorbance of the solution was recorded at 415 nm. The H_2_O_2_ content was calculated according to a standard curve of H_2_O_2_ ranging from 0 to 10 µM.

### Malondialdehyde Assay

Leaves were taken for determination of malondiaklehyde (MDA) content. Briefly, 200 mg of leaves were homogenized under liquid nitrogen, hydrated in 5 ml of 0.1% (w/v) trichloroacetic acid solution (TCA) and centrifuged at 12,000 g at 4°C for 10 min. One ml of the supernatants was reacted with 4 ml of 0.5% (w/v) thiobarbituric acid (TBA) solution containing 20% (w/v) TCA. The mixture was incubated at 100°C for 30 min and centrifuged at 12,000 g at 4°C for 10 min; the absorbance of the supernatant was read at 532 and 600 nm. MDA content was estimated by using extinction coefficient of 155 (mmol/L/com) [34].

### Evaluation of Abiotic Stresses Tolerance at the Booting Stage

For different abiotic stress tolerance evaluation at the booting stage, wild-type plants and four T_2_ *OsAPX2*-OX transgenic lines with stable *OsAPX2* over expression were grown in pots (25 cm diameter ) with soil in greenhouse at 25/19°C (day/night). When the flag leaf was just visible (about 10 days before heading), different treatments were performed. For drought treatment, water was withheld for 20 days. Then all the pots were rewatered simultaneously and left there until the plants reached maturity. For salt treatment, the pots were immersed in 200 mM NaCl for 7 days, and then were watered every day with 1 liter tap water. For cold treatment, the pots were transferred to a phytotron maintained at 14°C for 7 days, then moved to greenhouse at 25/19°C (day/night) and left there until the plants reached maturity. Stress tolerance was evaluated on the basis of mean spikelet fertility.

### Statistical Analysis

All the experiments were carried out in triplicate. The values shown in the figures are mean values±SD. For multiple comparisons, means were compared by one-way analysis of variance and Duncan’s multiple range test with a 5% level of significance.

## Results

### Isolation of a PEG-sensitive T-DNA Insertion Mutant

**Figure 1:**
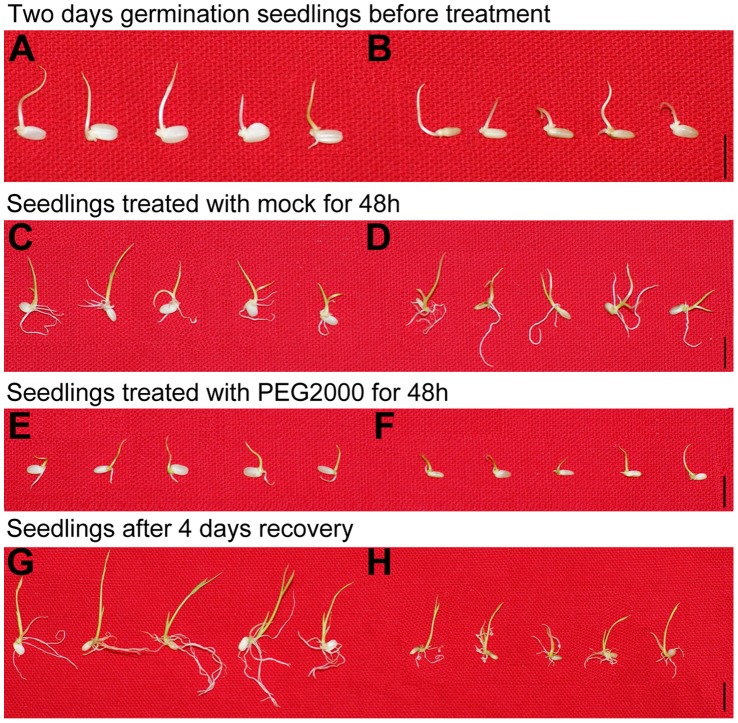
The phenotype of the PEG-sensitive T-DNA insertional rice line. After two days germination, wild-type (WT) (A) and mutant lines (B) were treated with 20% PEG2000. WT and mutant lines were treated with mock (C and D) and 20% PEG2000 (E and F) respectively, for 48 h. Seedlings of treated WT (G) and mutant seedlings (H) were recovered for 4 days. Bar = 1 cm.

**Figure 2:**
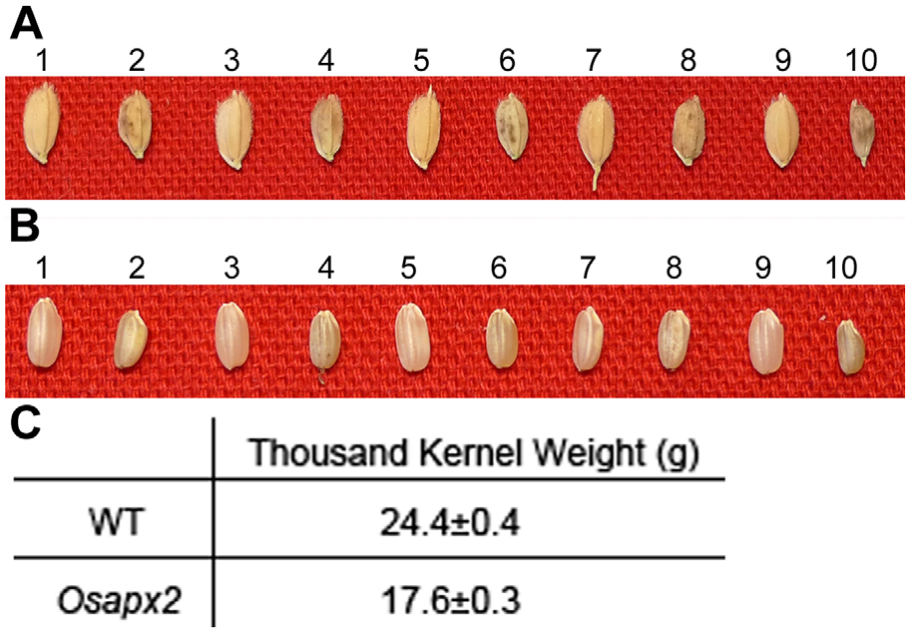
The seed phenotype of WT and *Osapx2*. A,B: the seed phenotype of WT (odd numbers) and *Osapx2* (even numbers), before dehulling (A) and after dehulling (B). C: The thousand kernel weight of WT and *Osapx2* seeds.

In an attempt to obtain drought sensitive rice mutants, we conducted genetic screening of a T-DNA insertion population containing more than 100,000 individual lines using 20% PEG2000. A PEG-sensitive mutant was identified with the phenotype of severe inhibition in the elongation of both shoots and roots (Figure 1). Additionally, the mutant seeds were smaller than the wild-type (Figure 2-A,B). As measured by the thousand kernel weight (TKW) of seeds, the mutant TKW was 17.6±0.3 g while that of wild-type was 24.4±0.4 g (Figure 2-C).

**Figure 3:**
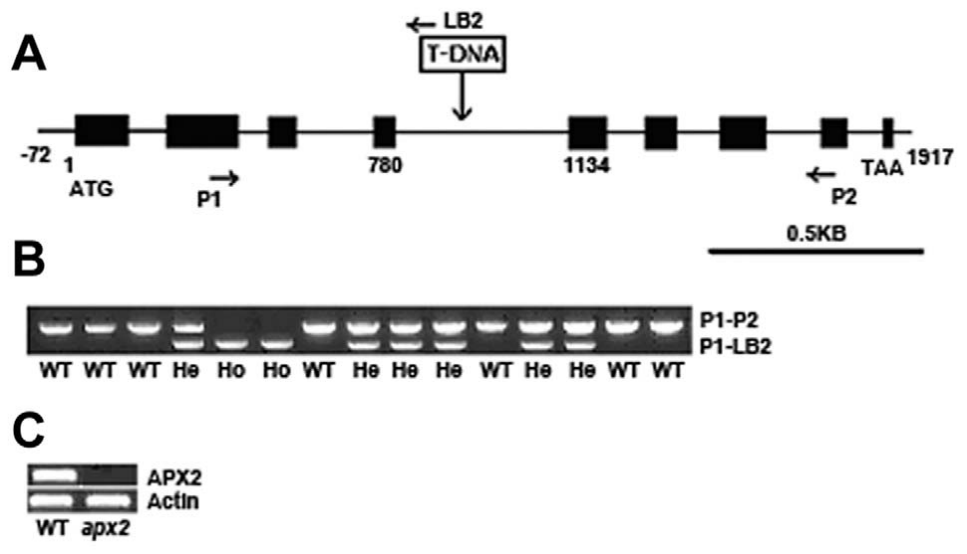
Molecular analysis of *Osapx2* mutants. A: Structure of *OsAPX2* and the location of T-DNA insertion. Black boxes indicate protein-encoding exons. Arrows indicate the locations of PCR primers used. B: Confirmation of co-segregation of the T-DNA insertion with the PEG-sensitive and small seed phenotype in leaves of T_1_ heterozygous progeny. Two PEG-sensitive seedlings (Ho; Homo type), PCR-amplified product only from the P1-LB2 primer set (*Osapx2*/*Osapx2*); six normal seedlings (He; Hetero type), amplified with both the P1-LB2 and P1–P2 primer sets (*Osapx2*/*OsAPX2*); and seven normal seedlings (WT), which amplified only with the P1–P2 primer set (*OsAPX2*/*OsAPX2*). C: RT-PCR expression analysis in leaves of wild-type phenotype and PEG-sensitive mutant seedlings. *Actin* gene is used as control.

T-DNA insertion of the mutant was confirmed by amplification of genomic fragment flanking the T-DNA insertion site using PCR walking assay [25]. BLAST search of the T-DNA flanking sequence against the rice genome database (http://blast.ncbi.nlm.nih. gov/Blast.cgi) identified that T-DNA was inserted into the fourth intron of ascorbate peroxidase 2 gene (*OsAPX2*, Os07 g0694700) (Figure 3-A). The full genome sequence of the *OsAPX2* spans approximately 2,553-bp on chromosome 7, contains nine exons and eight introns.

In order to test the co-segregation of PEG sensitivity and T-DNA insertion in T_1_ segregating population, we analyzed more than 100 individual plants using gene-specific PCR primers (P1 and P2) flanking the T-DNA insertion in *OsAPX2* and primer LB2 to the T-DNA left border (Figure 3-A). Results showed that the PEG- sensitive phenotype and small seed size phenotype was co-segregated with the homozygous T-DNA insertion (Figure 3-B). RT-PCR analysis showed that *OsAPX2* gene expression was completely abolished in the mutant (Figure 3-C).

### *OsAPX2* is Important for Growth and Development in Rice

**Figure 4:**
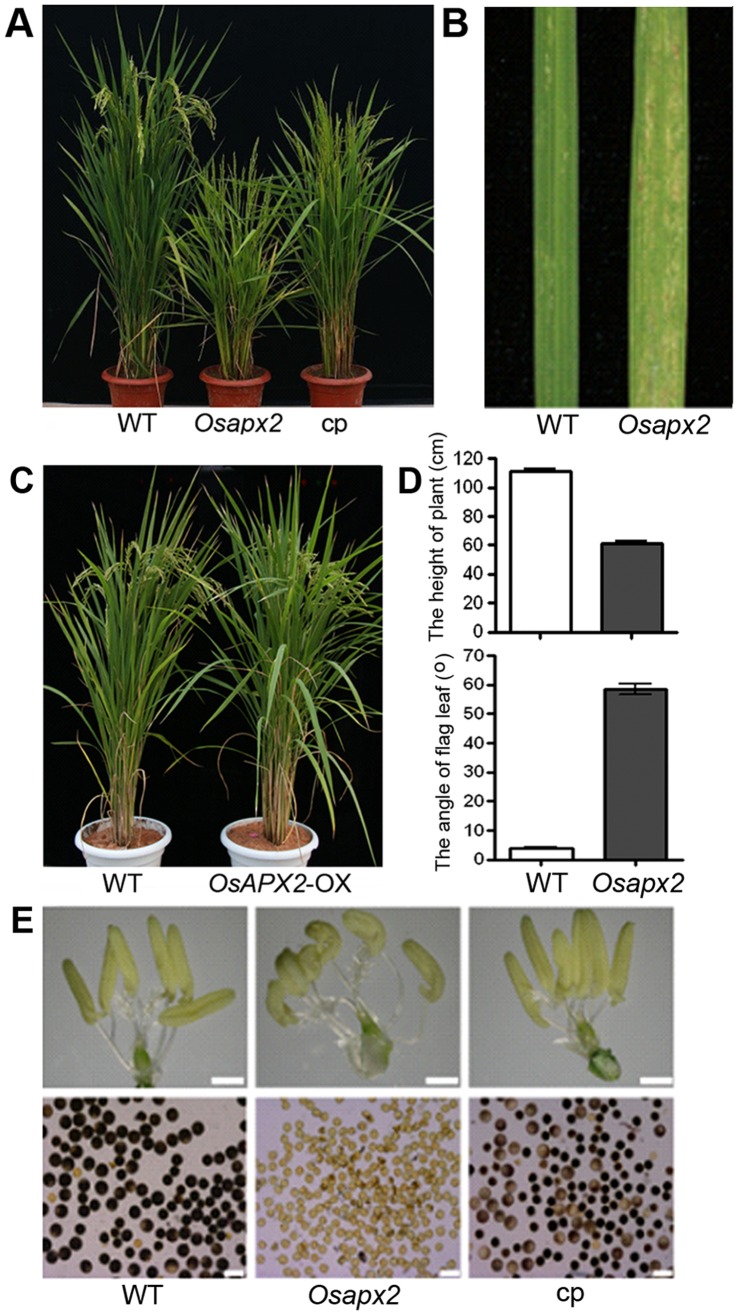
Characterization of wild-type, *Osapx2*, complementation and *OsAPX2*-OX plants. A,C: The phenotype of wild-type (WT), *Osapx2*, complementation (cp) plants and *OsAPX2*-OX plants at mature stage. B: The leaves of wild-type (WT), *Osapx2* (m) and complementation plants (cp) during the vegetative development stage. D: The comparison of the height and the flag leaf angle between wild-type and *Osapx2* mutant plants at the mature stage. E: Comparison of anther morphology and pollen viability stained with KI among wild-type (WT), *Osapx2* and complementation plants (cp). Bar = 20.0 µm.

Not only were the seeds of the *Osapx2* mutant smaller than the wild-type plants, but also the mutant plants were shorter than the wild-type plants. At the heading stage, the height of the mutant seedlings was about two-third of the wild-type Nipponbare (63 cm compared with 110 cm of wild-type) (Figure 4-A,D). The angle of flag leaf was increased in the mutant plants (Figure 4-D). Seedlings of the *Osapx2* mutants grown both in greenhouse and paddy field showed a lesion mimic phenotype in the middle of the leaf blade during the tillering stage. After the tillering stage, the lesion spots spreaded to the whole leaves in the mutant plants (Figure 4-B). The mutant plants were completely sterile which was supported by abnormal anther morphology and defective pollen viabiligy (Figure 4-E). Genetic analyses of T_1_ heterozygous progeny revealed that a segregation ration of wild-type (145):sterility (55) phenotype was 3∶1 (χ^2^ = 0.67< χ^2^_0.01,1_), suggesting that the mutated phenotype was controlled by a single recessive nuclear locus.

**Figure 5:**
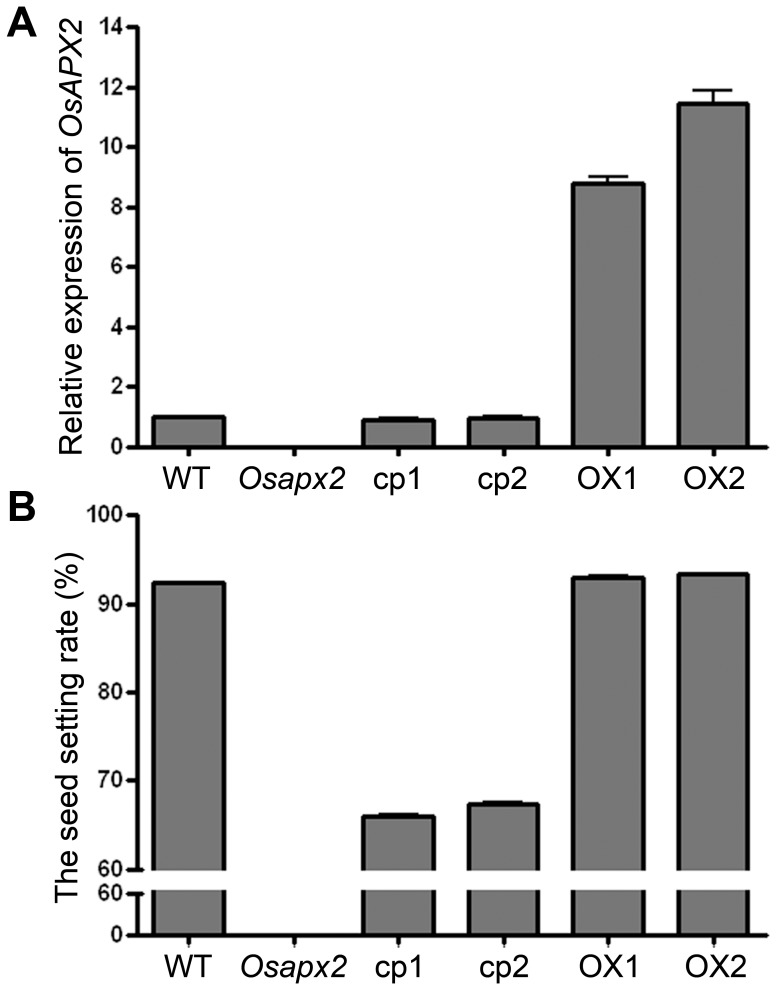
The expression of *OsAPX2* and seed setting rate in wild-type, *Osapx2*, complementation and *OsAPX2-*OX plants. A: RT-qPCR analysis of *OsAPX2* expression in wild-type (WT), *Osapx2*, complementation plants (cp1 and cp2) and *OsAPX2*-OX plants. B: Seed setting rates of wild-type (WT), *Osapx2*, complementation plants (cp1 and cp2) and *OsAPX2*-OX plants. Values represent means ± SD of three replicates.

Since the homozygous mutants were male sterile, the small seeds segregated from the heterozygous lines were used for complementary test. Calli derived from homozygous *Osapx2* mutant seeds were confirmed by PCR and transformed with *pOsAPX2::OsAPX2*, a construction of the full-length cDNA of *OsAPX2* driven by its own promoter. Among all the fifty-one transgenic plants obtained, thirty five plants showed the wild-type expression level of *OsAPX2* gene as determined by RT-qPCR (Figure 5-A) and recovered the wild-type phenotype in terms of seedling height, leaf appearance, anther shape, pollen viability and seed setting rate (Figure 4-A,E; Figure 5-B).

*OsAPX2* over expression construct driven by actin promoter was introduced into wild-type rice cultivar Nipponbare via *A. tumefaciens* mediated transformation. Totally more than 200 individual transgenic lines were generated and confirmed by PCR detection of the transgene. *OsAPX2* expression was examined by RT-qPCR of total RNA extracted from leaves of each transgenic line. *OsAPX2* expression was much higher in transgenic lines relative to wild-type plants (Figure 5-A). The *OsAPX2* transgenic plants have the same phenotype as the wild-type seedlings in which they had a similar seed setting rate (Figure 4-C, Figure 5-B).

### *OsAPX2* Expression is Spatially and Developmentally Regulated

**Figure 6:**
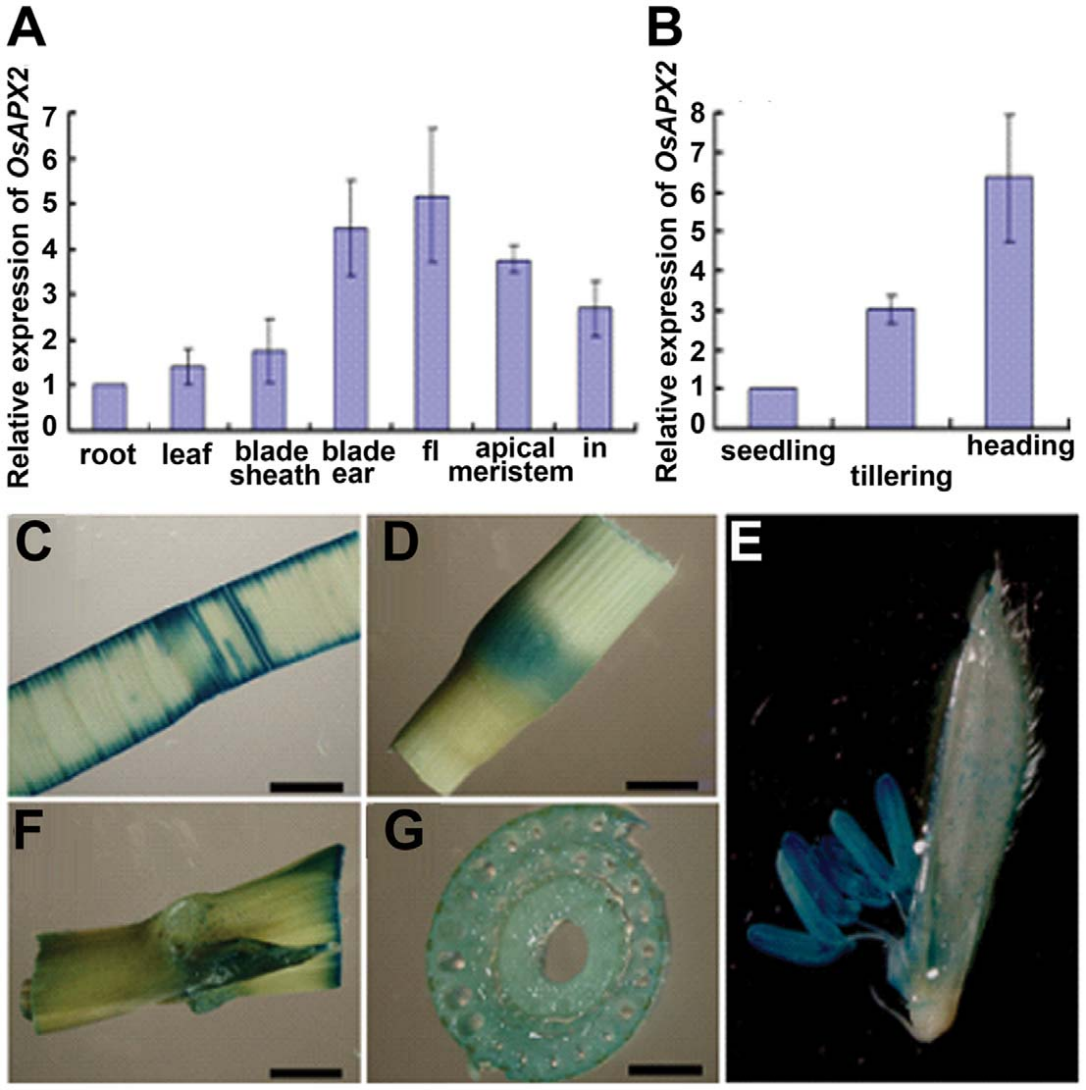
Developmentally regulated *OsAPX2* expression in rice tissues. A: RT-qPCR was performed to analysis the expression of *OsAPX2* in root, leaf, blade sheath, blade ear, flower (fl), apical meristem and internode (in). B: The expression of *OsAPX2* in leaves at different stages. C,D,E,F,G Expression patterns of *OsAPX2* revealed by GUS staining. Leaf (D), internode (D), blade ear (F) and the transversely side of internode (G), flower (E). Bar = 200 µm.

RT-qPCR was performed to analyze the expression pattern of *OsAPX2* in different tissues and in leaves at different developmental stages. As show in Figure 6-A, the expression of *OsAPX2* was low in root, leaf and blade sheath but high in blade ear, flower, apical meristem and internode. To further examine the expression pattern of *OsAPX2* comprehensively, the pCAMBIA1391Z vector with the fusion of the *OsAPX2* pormoter (from −2111 bp to −1 bp) and the GUS coding sequence was transformed into calli in the wild-type background by *Agrobacterium tumefaciens*-mediated transformation. Strong GUS activity was detected in organs including young leaf (Figure 6-C), internode (Figure 6-D) and blade ear (Figure 6-F), stem (Figure 6-G) and anther (Figure 6-E), which are consistent with above RT-qPCR results. Then we compared the gene expression at different developmental stages using leaves at seedling and tillering stages, and flag leaves at heading stage. Results showed that the expression of *OsAPX2* was higher in mature leaves than young leaves; *OsAPX2* was most strongly expressed in flag leaves at heading stage (Figure 6-B).

### *OsAPX2* Expression is Induced by Drought, Cold and Salt Stresses

**Figure 7:**
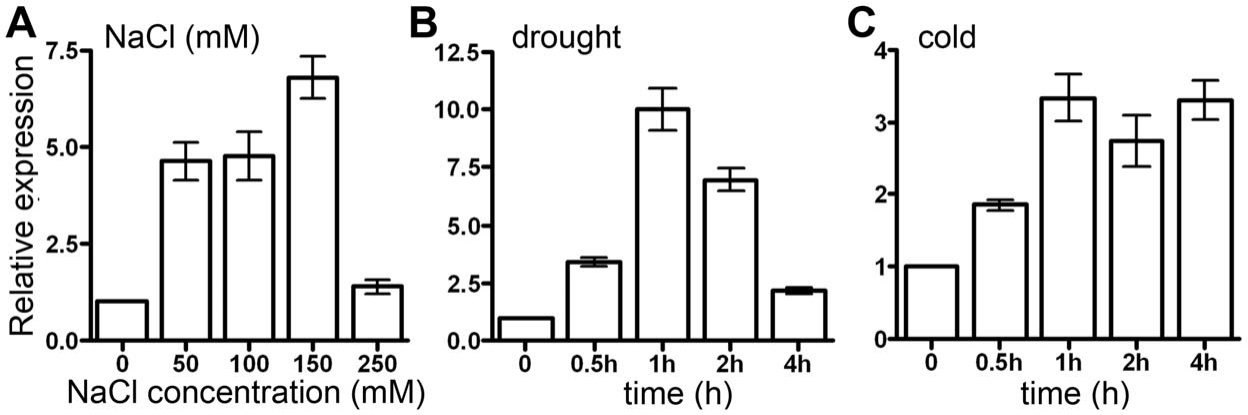
*OsAPX2* expression response to drought, cold and salt stresses. A: *OsAPX2* expression response to salt stress. B,C: Time course of *OsAPX2* expression during drought and cold treatments. *Actin* was used as an internal control. Data represent means ± SD of three replicates.

According to previous reports, the expression of *APX* genes is modulated by different environmental stimuli. To analyze the expression pattern of *OsAPX2* in response to abiotic stresses, 2-week-old rice plants were subjected to drought, cold (4°C) and salt stresses, respectively. The accumulation of the transcripts was determined by RT-qPCR. As shown in Figure 7-A, *OsAPX2* expression was induced by salt stress. After salt treatment for 1 h, transcript accumulation of *OsAPX2* was increased by 4.5-fold under the treatment of 50 mM and 100 mM NaCl compared with the water mock control. Gene expression was increased by 7-fold after 150 mM NaCl treatment but was reduced to control level when treated with 250 mM NaCl. Also, *OsAPX2* expression was induced by drought treatment. As shown in Figure 7-B, the expression of *OsAPX2* reached peak at 1 h treatment but gradually reduced after 2 h treatment. Similarly, *OsAPX2* expression was induced within 0.5 h after cold treatment and was highest after 1 h treatment (Figure 7-C).

**Figure 8:**
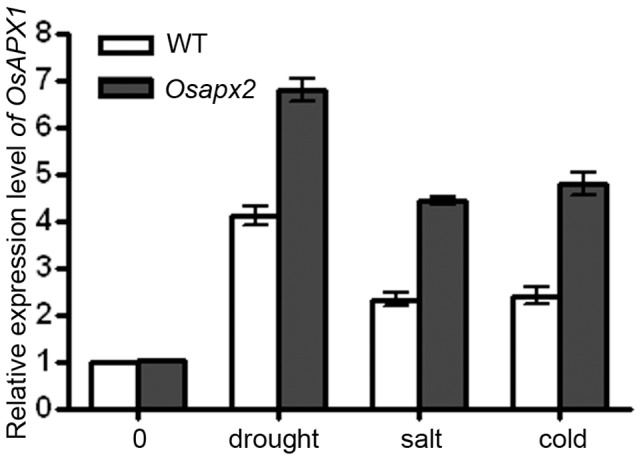
The expression of *OsAPX1* in *Osapx2*. The expression of *OsAPX1* was detected in leaves of wild-type and *Osapx2* mutant seedlings under normal condition and after stress treatments. Data represent means ± SD of three replicates.

Since there are two cAPX genes in rice genome and OsAPX1/OsAPX2 proteins were 83% identity to each other. The expression profile of *OsAPX1* was analyzed in both wild-type and indentified *Osapx2* mutant seedlings under various treatments. As shown in Figure 8, the expression pattern of *OsAPX1* induced by different stresses is similar in wild-type and *Osapx2*, while the expression of *OsAPX1* induced more notably in *Osapx2* mutant than wild-type by different stress treatments.

### Expression of *OsAPX2* is Critical to Abiotic Stress Tolerance

**Figure 9:**
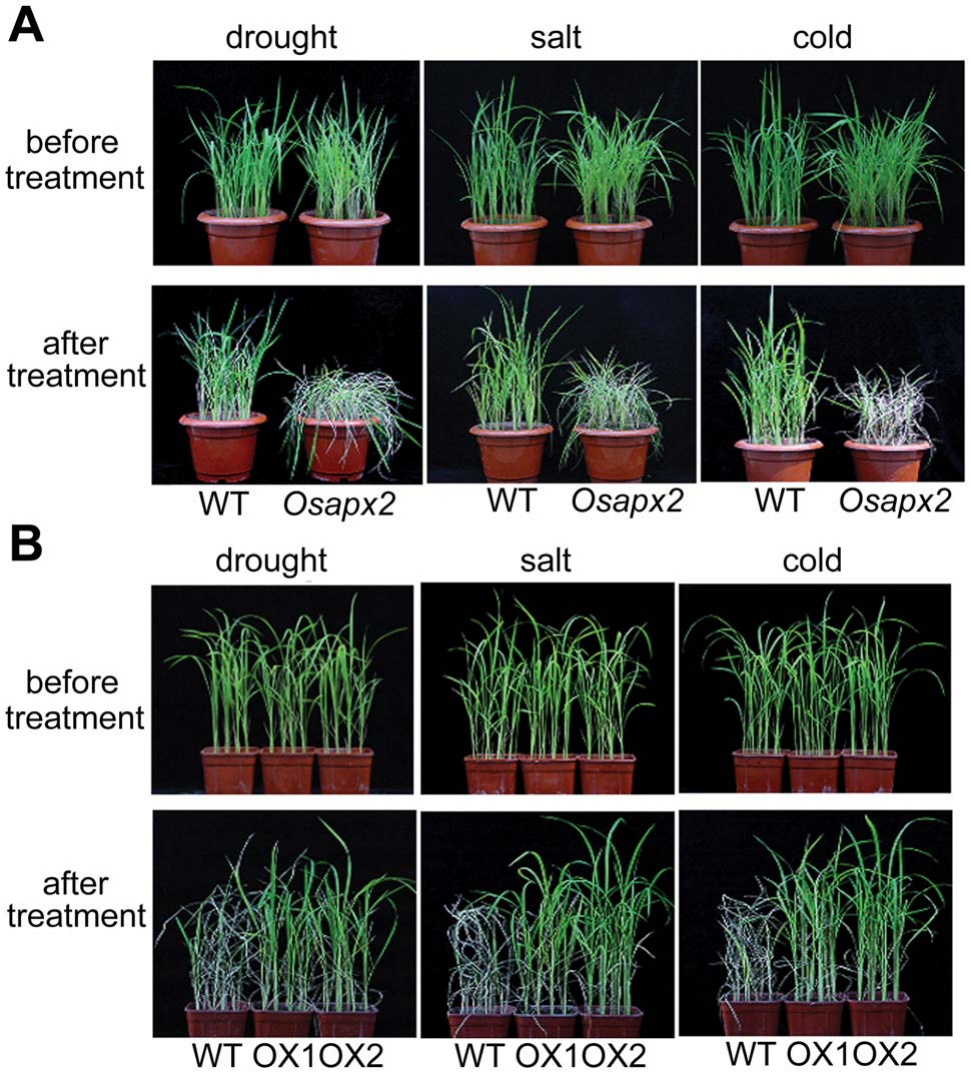
Effect of *OsAPX2* expression on drought, salt and cold tolerance in rice. A: Without *OsAPX2*, *Osapx2* mutant plants showed sensitive to drought, salt and cold treatments. B: *OsAPX2*-OX transgenic plants showed tolerance to drought, salt and cold treatments.

The induction of *OsAPX2* expression by abiotic stresses suggests *OsAPX2* might function in stress tolerance in rice. In order to demonstrate the role of *OsAPX2* in abiotic stresses tolerance, we compared the growth of *Osapx2* mutant, T_1_ *OsAPX2*-OX lines and wild-type plants under drought, salt and cold stresses. As shown in Figure 9, *Osapx2* mutant plants were more sensitive to drought stress than its segregated wild-type plants when water withheld for 6 days (Figure 9-A). By contrast, *OsAPX2*-OX plants were more tolerant to drought stress than the wild-type plants; they were growing well even after water was withheld for 10 days (Figure 9-B).

Similarly, *Osapx2* mutant plants were more sensitive to salinity stress than the wild-type plants (Figure 9-A), while *OsAPX2*-OX lines were more tolerant to salt stress than the wild-type plants (Figure 8-B). When treated with 200 mM NaCl for 10 days, *Osapx2* mutant plants were almost dead, and the growth of the wild-type plants was inhibited. Wild-type plants were beginning bleaching after salt stress for 18 days, while *OsAPX2*-OX seedlings were still green and growing well (Figure 9-B). Moreover, *Osapx2* plants were more sensitive to cold stress than the wild-type plants (Figure 9-A), whereas *OsAPX2*-OX plants were more tolerant to cold stress than wild-type plants. When exposed to 16°C in a chamber for 14 days, *Osapx2* mutant plants were becoming wilting, and the growth of the wild-type was inhibited. After cold treatment for 18 days, wild-type seedlings were becoming wilting, while *OsAPX2*-OX seedlings were still growing well (Figure 9-B).

**Figure 10:**
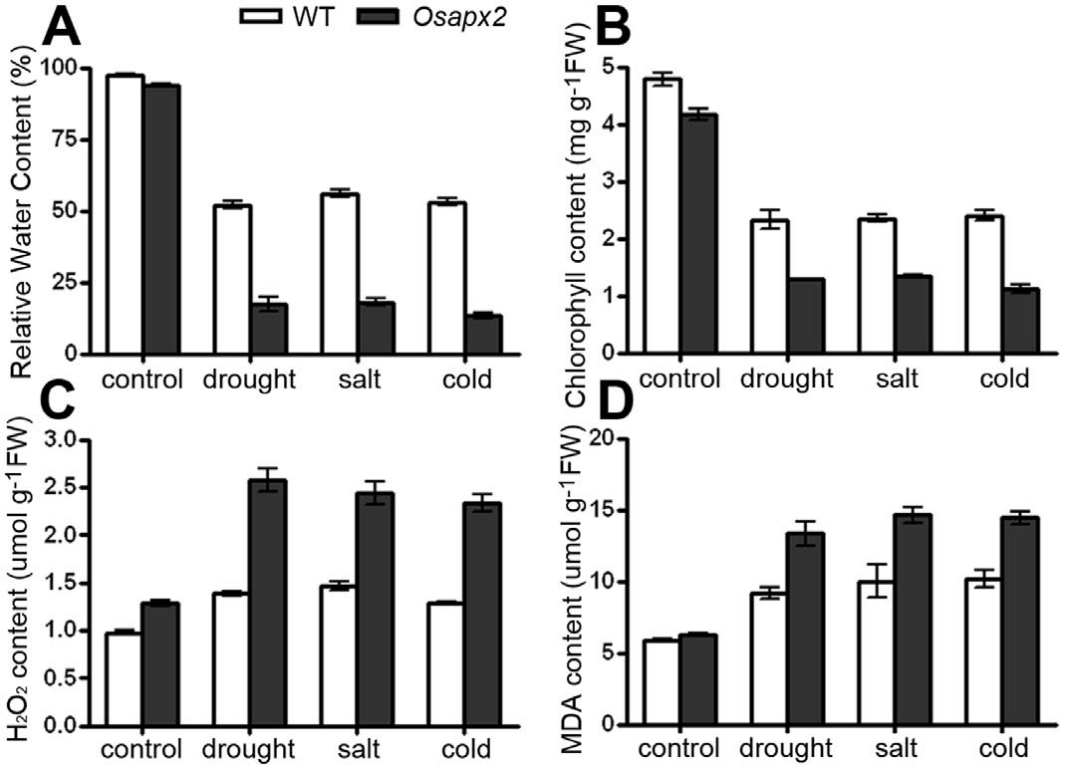
The effects of stress treatments on wild-type and *Osapx2* plants. A: Relative water content. B: Chlorophyll content. C: H_2_O_2_ content. D: MDA content. Values represent the mean ± SD of three replicates.

**Figure 11:**
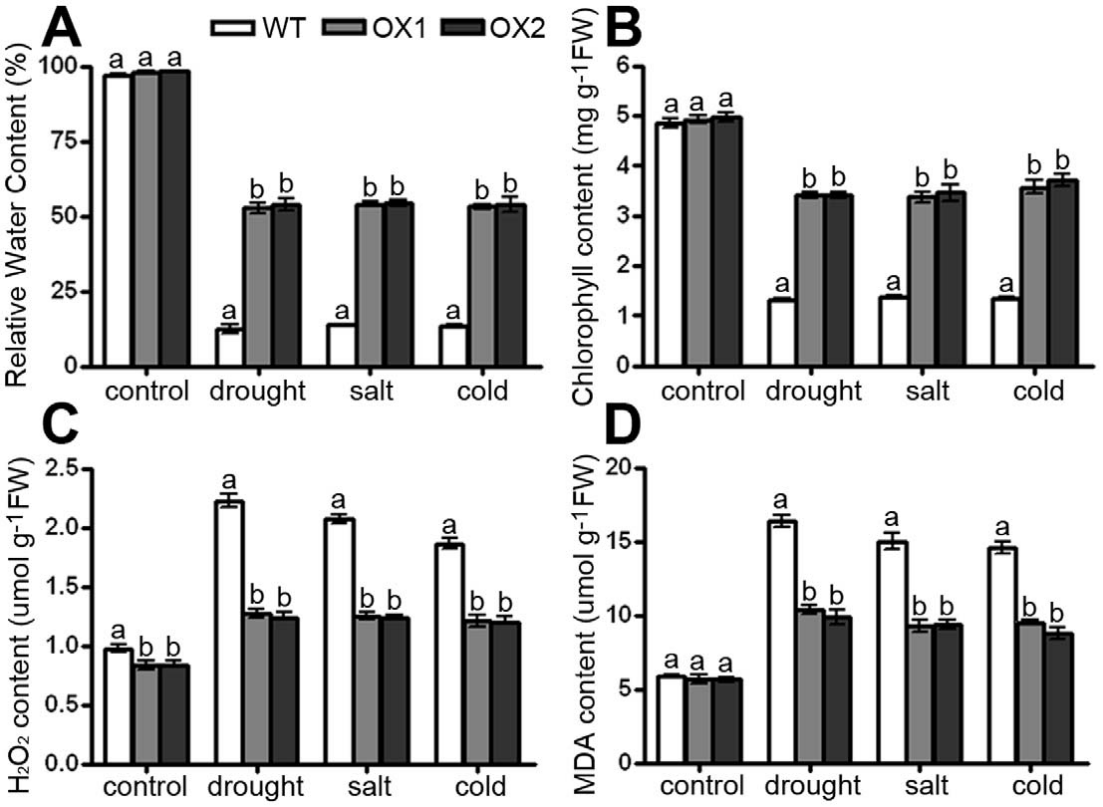
The effects of stress treatments on wild-type and *OsAPX2-*OX plants. A: Relative water content. B: Chlorophyll content. C: H_2_O_2_ content. D: MDA content. Data are mean of three replicates and were compared by one-way analysis of variance and Duncan’s multiple range test. Different letters (a–b) indicate significant differences (*P*<0.05) between lines.

The responses of *Osapx2* mutant, wild-type and *OsAPX2*-OX plants to abiotic stresses are consistence to their relative water content, chlorophyll content, H_2_O_2_ and MDA contents. The abiotic stress sensitive phenotype of *Osapx2* was consistent with more water loss, lower chlorophyll content, higher H_2_O_2_ and MDA contents after stress treatments (Figure 10). By contrast, the abiotic stress tolerance phenotype of *OsAPX2*-OX was consistent with less water loss, higher chlorophyll content, and lower H_2_O_2_ and MDA contents (Figure 11).

### Overexpression of *OsAPX2* Improved Spikelet Fertility in Rice under Abiotic Stresses

**Figure 12:**
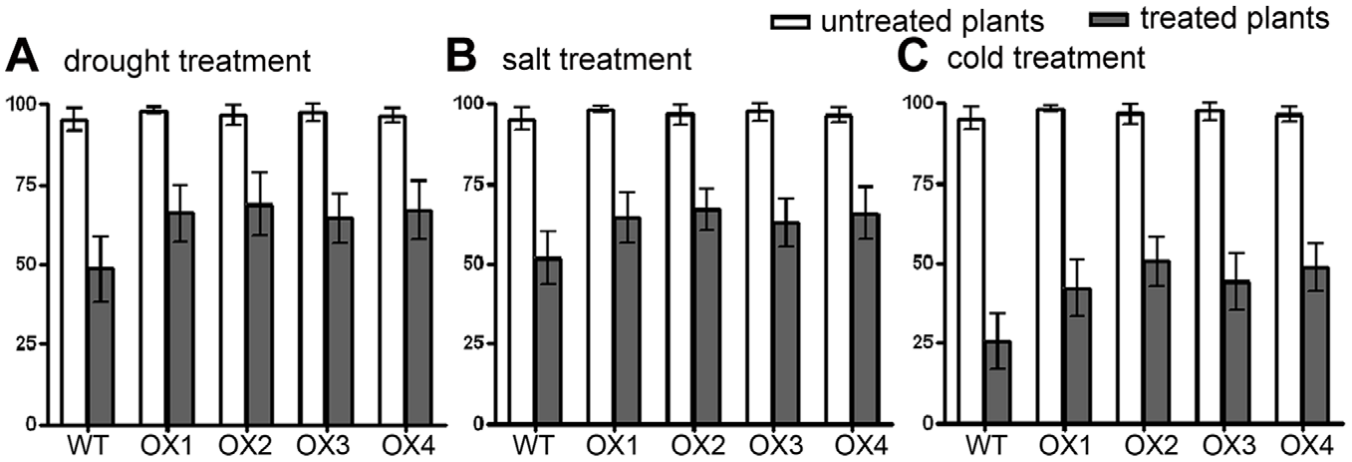
Spikelet fertility after stress treatments in T_2_ *OsAPX2*-OX transgenic plants and WT plants. OX1, OX2, OX3 and OX4 are independent *OsAPX2*-OX lines. A: drought treatment, B: salt treatment, C: cold treatment. Values represent the mean ± SD (*N* = 10).

Studies have shown that rice yields are affected by the abiotic stress at the booting stage [35]. In order to understand whether or not the *OsAPX2* functions in improving rice yields through enhancing tolerance to abiotic stresses, we compared the spikelet fertility of both *OsAPX2*-OX and wild-type plants at the booting stage. The T_2_ transgenic *OsAPX2*-OX plants and wild-type plants were stressed under drought, salt and low temperature treatments at the booting stage, respectively. Four independent *OsAPX2-OX* lines (OX1, OX2, OX3 and OX4) were used for each treatment in our assays. As shown in Figure 12, *OsAPX2*-OX and wild-type plants had similar spikelet fertilities under normal condition, while significant differences were found between *OsAPX2-OX* transgenic lines and wild-type plants under stress treatments. For drought treatment, the spikelet fertilities were decreased by 75% in wild-type plants, but were decreased by 56%, 52%, 60% and 57% in *OsAPX2*-OX lines (OX1, OX2, OX3 and OX4, respectively; Figure 12). For salt treatment, the spikelet fertilities were decreased by 54% in wild-type plants, but only reduced by 38%, 33%, 39% and 37% in *OsAPX2*-OX lines (OX1, OX2, OX3 and OX4, respectively; Figure 12). For cold treatment, the spikelet fertilities were decreased by 76% in wild-type plants, but only were reduced by 66%, 59%, 64% and 67% in *OsAPX2*-OX lines (OX1, OX2, OX3 and OX4, respectively; Figure 12). Moreover, our experiments in drought shed obtained the same results, showing that *OsAPX2*-OX plants were more tolerant to drought stress than wild-type plants at the booting stage (Figure S1).

### OsAPX2 is Essential for Scavenging H2O2 in Rice

**Figure 13:**
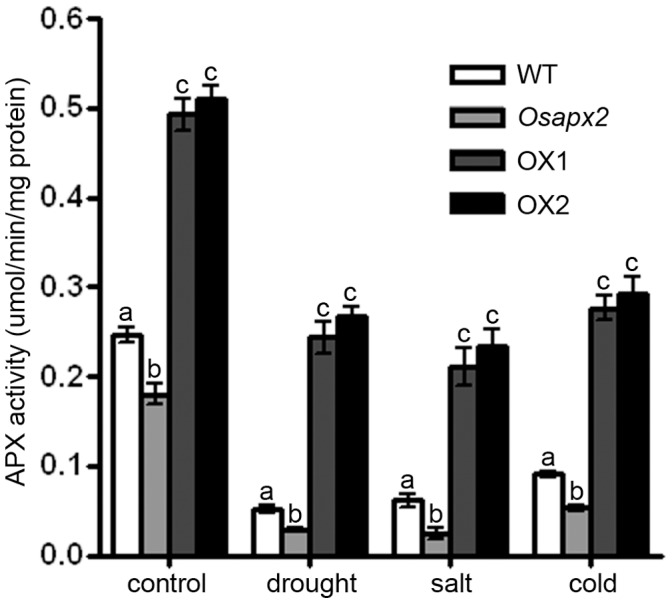
APX activity assay of wild-type, *Osapx2* and *OsAPX2*-OX plants. Five leaf seedlings grown in the half-strengthened MS liquid medium were treated with different treatments. For salt treatment, wild-type, *Osapx2* and *OsAPX2*-OX plants were treated with 150 mM NaCl for 24 h; for cold treatment, plants were transferred to a 10°C incubator for 24 h; for drought treatment, plants were transferred to dry sterile petri dishes or 2 h. Different letters (a–c) indicate significant differences (*P*<0.05) between lines.

In order to understand whether or not APX2 is important in scavenging H_2_O_2_ in rice, we analyzed the APX activity and H_2_O_2_ contents in wild-type, *Osapx2* and *OsAPX2*-OX plants. As shown in Figure 13, APX activity in *Osapx2* was lower than that of wild-type plants under both normal and stress conditions; by contrast, *OsAPX2*-OX transgenic lines showed higher levels of APX activity under both normal and stress conditions compared to the wild-type plants.

**Figure 14:**
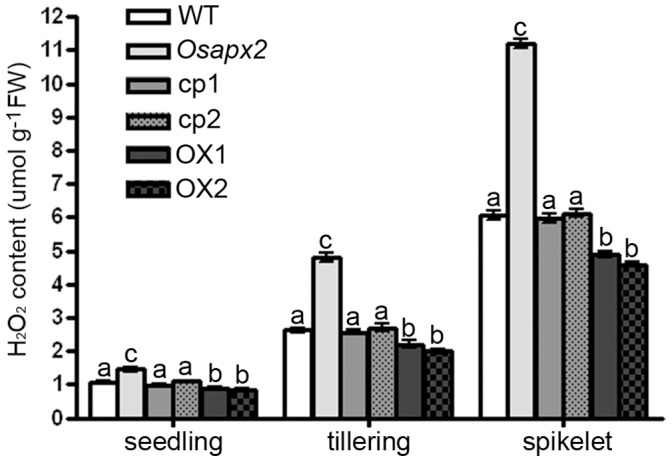
H_2_O_2_ content assay of wild-type, *Osapx2*, complementation and *OsAPX2*-OX plants at different developmental stages. H_2_O_2_ content assay was performed in wild-type, *Osapx2*, independent complementation lines (cp1 and cp2) and independent *OsAPX2*-OX lines (OX1 and OX2) at different developmental stages. Different letters (a–c) indicate significant differences (*P*<0.05) between lines.

H_2_O_2_ content was analyzed in wild-type, *Osapx2*, complementation lines and OsAPX2-OX lines at different developmental stages. As shown in Figure 14, for all of the lines, the H_2_O_2_ level was lower in seedlings, but increased gradually with the growth and reached a high level in spikelets. However, H_2_O_2_ level of *Osapx2* mutant plants was significantly higher than that of wild-type in all growing stage. H_2_O_2_ content of complementation lines was similar to that of wild-type plants at seedling stage, while it is still higher than that of wild-type plants at tillering stage and in spikelet. By contrast, the accumulation of H_2_O_2_ in *OsAPX2*-OX transgenic lines was lower than the wild-type plants.

## Discussion

### OsAPX2 is a Central Player in Rice Growth and Abiotic Stress Tolerance

In this study, using both the *Osapx2* knockout mutants and the *Osapx2* overexpression lines, we demonstrated that *OsAPX2* plays an important role in tolerance to abiotic stresses in rice. In addition, we found that the *Osapx2* knockout mutants exhibited a pleiotropic phenotype including semi-dwarf, severe leaf minic lesion and male-sterility (Figure 4-A,E). Our results indicate that *OsAPX2* not only functions in protecting rice seedlings from abiotic stresses but also is a central player in the growth and development in rice.

The fact that knocking out the *Osapx2* gene causes severe growth defects and abiotic stress responses indicates that *Osapx2* gene is a central component of the reactive oxygen gene network in rice. On the other hand, this also shows that the function of *Osapx2* gene is not compensated by other members of the APX gene family in rice [15]. This might be true since Rosa et al. (2010) found that silencing of *OsApx2* reduced *OsApx1* and *OsApx7* expression [17]. Similarly, knocking out the *Atapx1* gene does not lead to an increase in the expression of the other APX isozymes in the APX gene family in *Arabidopsis* [10]. These may suggest that a more complicated compensation system exists in plants. The APX reactive oxygen scavenging network might be compensated and cooperated by other system(s) outside the APX gene family.

### OsAPX2 Plays a Critical Role in Protecting the Functions of Chloroplasts in Rice

Studies from *Arabidopsis* have shown that the cytosolic ascorbate peroxidase APX1 plays a key role in ROS homeostasis in cells under abiotic stresses. Davletova et al. (2005) reported that in the absence of APX1, the entire chloroplastic H_2_O_2_-scavenging system collapsed, H_2_O_2_ levels increased, and protein oxidation occurred, indicating that APX1 plays a critical role in cross-compartment protection of chloroplast functions [36]. This study implies that the cytosolic ascorbate peroxidase APX1 functions as a defense barrier between the three major ROS-producing organelles of plant cells (i.e., the chloroplast, mitochondria, and peroxisomes). *OsAPX2*, a cytosolic ascorbate peroxidase in rice, shares the highest degree of similarity with the *Arabidopsis* APX1 in all 8 members of the rice APX gene family (Figure S2). Our results have shown that *Osapx2* mutants were sensitive to abiotic stresses (Figure 9-A). Interestingly, *Osapx2* mutants had a significant low level of chlorophylls under abiotic stresses relative to the wild-type plants (Figure 10-B). By contrast, over expression of *OsAPX2* increased the chlorophyll contents to a level higher than the wild-type plants (Figure 11-B). These results suggest that the rice cytosolic ascorbate peroxidase OsAPX2 might also function as a defense barrier in cellular oxidative stress and plays a key role in cross-compartment protection of chloroplast functions in rice.

### APXs Might Control Plant Growth and Development through the H_2_O_2_ Signal Transduction Pathway

Our results showed that *OsAPX2* disruption inhibited seedling growth and anther development (Figure 4-A,E). Previous studies found that silencing of either *OsAPX1* or *OsAPX2* gene produced a semi-dwarf phenotype [17], and low temperatures caused male sterility in rice [18]. *Arabidopsis* APX1 knockout mutant also had a suppressed growth and development phenotype [10]. However, regulation mechanism of plant growth and development by APX genes remains unclear. Since APX function to remove cellular H_2_O_2_ and disturbing AXP activity enhances H_2_O_2_ production in plants, therefore, it is reasonable to hypothesize that APXs might control plant growth and development through the H_2_O_2_ signal transduction pathway. H_2_O_2_ is a signaling molecule and plays versatile roles in plants [37]. Studies have shown that H_2_O_2_ regulates the expression of many transcription factors, including ZAT zinc finger transcription factor, WRKY, heat shock transcription factor, and ethylene response factor [10], [36], [38]. It is possible that H_2_O_2_ might affect plant growth and development through regulating the expression of these transcription factors. Indeed, knocking out AtAPX1 gene elevated the levels of ZAT zinc finger transcription factor, WRKY, heat shock transcription factor, and ethylene response factor [10]. Moreover, the expression of a flowering related gene GIGANTEA in *Arabidopsis* was elevated in *AtAPX1* knockout mutants. But there is no direct link between the GIGANTEA expression and the rise in H_2_O_2_ levels in *AtAPX1* mutants [10]. Therefore, more studies are needed to identify the transcription factor(s) and/or gene(s) involved in APX regulation of growth and development in plants.

## Supporting Information

Figure S1 **Drought stress experiments preformed in drought shed.** Black lines indicated *OsAPX2*-OX plants. Red lines indicated wild-type plants.
(TIF)

Figure S2 **Phylogenetic tree of rice and** ***Arabidopsis*** **APX proteins.** The tree was constructed with the DNAMAN tree program with amino acid sequences of *Arabidospis* APXs (APX1∼APX6, tAPX and sAPX) and OsAPXs (OsAPX1∼OsAPX8).
(TIF)
