# Supplementary figures and images for: Gene Knockout Study Reveals That Cytosolic Ascorbate Peroxidase 2(OsAPX2) Plays a Critical Role in Growth and Reproduction in Rice under Drought, Salt and Cold Stresses
Source: PLoS One. 2013 Feb 28;8(2):e57472. doi: 10.1371/journal.pone.0057472 (PMC3585366; doi:10.1371/journal.pone.0057472)

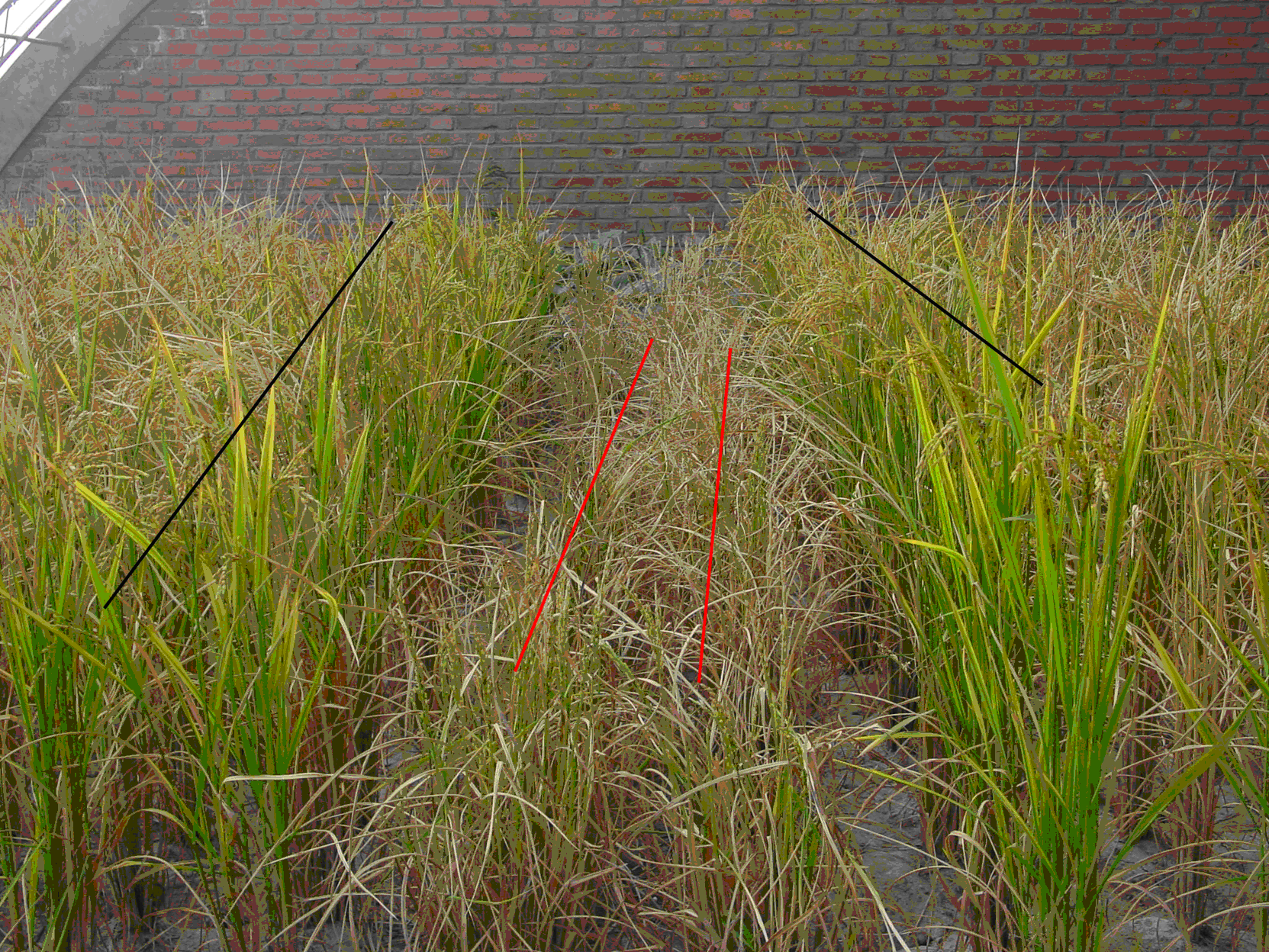

Supplement: Figure S1 — Drought stress experiments preformed in drought shed. Black lines indicated OsAPX2-OX plants. Red lines indicated wild-type plants. (TIF) [file pone.0057472.s001.tif]

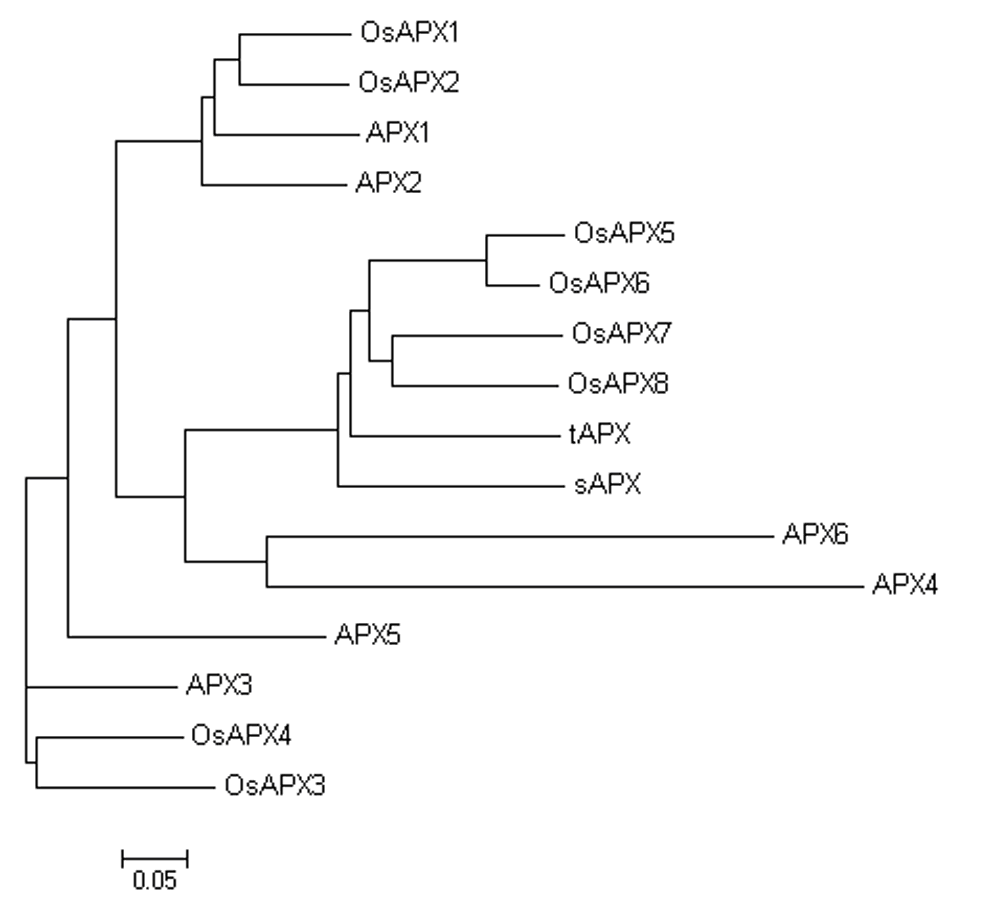

Supplement: Figure S2 — Phylogenetic tree of rice and Arabidopsis APX proteins. The tree was constructed with the DNAMAN tree program with amino acid sequences of Arabidospis APXs (APX1∼APX6, tAPX and sAPX) and OsAPXs (OsAPX1∼OsAPX8). (TIF) [file pone.0057472.s002.tif]
